# Characterization of *Enterococcus faecium* Based on Multi-Omics Approaches: Genomic, Transcriptomic, and Phenotypic Analyses

**DOI:** 10.3390/vetsci13010103

**Published:** 2026-01-21

**Authors:** Jiayan Huang, Haoyu Fan, Yurui Wang, Xiao Yue, Zixuan Li, Zhanchun Bai, Da Qiong, Zhuoma Gesang, Sizhu Suolang

**Affiliations:** 1 College of Animal Science, Xizang Agricultural and Animal Husbandry University, Linzhi 860000, China; 202200201099@stu.xza.edu.cn (J.H.);; 2 Linzhi Municipal Agriculture and Rural Affairs Bureau, Livestock and Veterinary Station (Animal Disease Control Center), Linzhi 860000, China; 3 Xizang Autonomous Region Animal Disease Prevention and Control Center, Lasa 850000, China

**Keywords:** yak, *Enterococcus faecium*, multi-omics

## Abstract

Animal-derived *E. faecium* poses a public health risk due to its capacity to acquire antimicrobial resistance (AMR) and virulence genes. However, the pathogenicity and cross-host transmission potential of strains originating from unique environments, such as the Qinghai–Tibet Plateau, remain poorly understood. In this study, a strain of *E. faecium* was isolated from yak feces. We constructed a phylogenetic tree and identified AMR and virulence genes via whole-genome sequencing. Antimicrobial susceptibility testing was performed to determine its resistance phenotype. An in vivo mouse infection model was established to assess pathogenicity, and transcriptomic analysis was utilized to investigate the host’s molecular response mechanisms in infected intestinal tissue. The results indicated that this yak-derived strain is closely related to human clinical isolates, suggesting a risk of cross-host transmission. The strain harbored the AMR genes *AAC(6′)-Ii*, *msrC*, and *eatAv* and exhibited resistance to penicillin, kanamycin, erythromycin, and clindamycin. The strain harbored key virulence genes, such as *bopD*, *Acm*, and *ClpP*. Infection with this strain caused characteristic inflammatory damage in mouse intestinal tissue, as revealed by histopathological examination, including epithelial necrosis, vascular congestion, and inflammatory cell infiltration. Transcriptomics further delineated a complete “Recognition–Response–Damage” signaling axis: pathogen recognition through Toll-like receptors and NOD-like receptors activates the NF-κB and MAPK signaling pathways. This activation is accompanied by significant upregulation of various inflammatory factors and recruits immune cells via chemokine signaling, ultimately leading to tissue damage. Our findings provide insights into the pathogenic pathway of this strain from genetic determinants to phenotypic manifestations, providing a theoretical foundation for assessing the public health risk posed by animal-derived *E. faecium* and for developing targeted intervention strategies.

## Simple Summary

*Enterococcus faecium* has emerged as a critical pathogen in hospital-acquired infections worldwide. This bacterium not only exhibits inherent resilience but also possesses a remarkable capacity to acquire antimicrobial resistance genes, posing a serious challenge to public health. Beyond clinical settings, *E. faecium* is widely distributed in animals and natural environments, serving as a reservoir for antimicrobial resistance genes and virulence factors, thereby contributing to persistent risks of cross-species transmission. In this study, a bacterial strain was isolated from yak feces collected on the Qinghai–Tibet Plateau and was characterized as *E. faecium*. We aim to systematically investigate its phylogenetic relationship with human-derived strains, its antimicrobial resistance phenotypes and genetic determinants, and its virulence potential through an integrated multi-omics approach encompassing genomics, transcriptomics, and phenotypic assays. Furthermore, we seek to fully elucidate the pathogenic signaling pathway from host recognition to tissue injury. Our findings provide critical evidence for assessing the public health risks associated with animal-derived *E. faecium* and offer novel theoretical insights into its pathogenic mechanisms.

## 1. Introduction

*Enterococcus faecium* is recognized globally as one of the “top ten pathogens” responsible for hospital-associated infections, posing a significant threat in clinical settings—particularly in the United States, where it ranks as the third leading cause of healthcare-associated infections [1,2]. This bacterium can cause several serious infections, including endocarditis, urinary tract infections, and bloodstream infections [3,4]. Studies have reported that bloodstream infections caused by vancomycin-resistant *E. faecium* are associated with significantly increased patient mortality [5]. Moreover, infection-related mortality is particularly pronounced among susceptible populations, such as immunocompromised individuals, the elderly, and children [6]. Even more concerning is the global spread of multidrug-resistant *E. faecium* strains, which further narrows the spectrum of available clinical treatment options and amplifies the challenges of infection control.

*E. faecium*, a widespread Gram-positive bacterium [7], exists in a variety of ecological niches, including humans and different animal species. This widespread presence creates a potential risk for cross-species transmission. The bacterium can establish stable colonization in the intestines of farm animals, such as pigs and cattle, as well as among companion animals like dogs and cats, and even in wildlife [8,9]. This “broad host range” contributes to its widespread distribution in both natural and anthropogenic environments. Multiple epidemiological studies have confirmed its cross-species transmission risk. For instance, investigations in the Thailand–Laos border region reported detection rates of *E. faecium* as high as 73.8–80.6% in pigs, pig carcasses, and retail pork samples, while the detection rate in concurrent human samples reached 67% [10]. This suggests the bacterium can spread via animal–environment–human pathways. In Bangladesh, researchers isolated antimicrobial-resistant *E. faecium* strains from food animals (e.g., pigs), confirming that animal-derived strains may enter human society through the food chain, posing a direct public health threat [11]. More critically, evidence indicates that *E. faecium* strains isolated from cattle feces share core virulence factors with human clinical isolates [12], implying that animal-derived strains possess the molecular foundation to cause infections across species in humans.

Given the clinical threat posed by *E. faecium*, the ongoing dissemination of antimicrobial resistance, and its risk of cross-species transmission, an in-depth investigation into the resistance profiles, virulence mechanisms, and transmission pathways of animal-derived *E. faecium* is of significant importance for developing targeted resistance control strategies and safeguarding public health. In recent years, advancements genomic sequencing technologies have enhanced our understanding of genetic backgrounds. For instance, a study conducted in Poland using whole-genome sequencing highlighted that *E. faecium* obtained from wild birds can act as a reservoir for antimicrobial resistance and virulence genes [13]. However, these single-omics approaches, which focus solely on static genetic profiles, are not sufficient for fully understanding the dynamic interactions between pathogens and hosts. Thus, there is a need to integrate multi-omics methods into a genomic framework. This study integrates whole-genome sequencing, transcriptome sequencing, and phenotypic experiments to systematically characterize an *E. faecium* isolate, aiming to explore its antimicrobial resistance, virulence factors, and infection mechanisms, which establishes a foundation for creating innovative anti-infection strategies.

## 2. Materials and Methods

### 2.1. Sample Source

For pathogen isolation, a fresh fecal sample was collected under aseptic conditions from a young yak (3 months old) with diarrhea in Jiali County, Naqu, during August 2023. The sampling method utilized a sterile swab inserted about 2 cm into the rectum. Subsequently, the swab was transferred to a tube containing sterile phosphate-buffered saline and delivered to the laboratory on ice.

### 2.2. Experimental Animals

In this study, we utilized Kunming mice of specific pathogen-free (SPF) grade, supplied by Sichuan Dashuo Experimental Animal Co., Ltd. (Chengdu, China). A total of ten 6-week-old mice, comprising five males and five females with a mean body weight of around 32 g, were acquired.

### 2.3. Primary Reagents and Instruments

Columbia Blood Agar Base was purchased from Qingdao Hope Bio-Technology Co., Ltd. (Qingdao, China). The 2× BenchTop™ Taq Master Mix enzyme was acquired from Accurate Biology (Hunan) Co., Ltd. (Changsha, China). The oligonucleotide primers were obtained from Sangon Biotech (Shanghai) Co., Ltd. (Shanghai, China), based on the designed sequences. The transmission electron microscope was obtained from Hitachi High-Tech Europe GmbH (Tokyo, Japan).

### 2.4. Bacterial Isolation and Identification

The fecal sample was streaked onto Columbia blood agar plates and then incubated under aerobic conditions at 37 °C for a period of 24 h. *Enterococcus* isolates were preliminarily identified to the genus level following the microbiological and biochemical methods described by Soltani S et al. [14], including the catalase test, growth in 6.5% NaCl, and hydrolysis on bile esculin agar. Subsequently, the species that had been confirmed were then transferred into Lysogeny Broth (LB, Qingdao Hope Bio-Technology Co., Ltd., Qingdao, China) supplemented with 20% glycerol and preserved at −80 °C for subsequent analysis.

### 2.5. Amplification of Target Genes and Analysis

Genomic DNA was extracted using the boiling method [15]. Polymerase chain reaction (PCR) amplification was performed with *E. faecium*-specific primers [16] to confirm the strain identity. The specific primer sequences, synthesized by Sangon Biotech (Shanghai, China) Co., Ltd., were as follows: forward primer FM1: 5′-GAAAAAACAATAGAAGAATTAT-3′; reverse primer FM2: 5′-TGCTTTTTTGAATTCTTCTTTA-3′. Amplifications were carried out in a total reaction volume of 25 μL. The mixture included 1 μL of both the forward and reverse primers (at a concentration of 10 μM each), 12.5 μL of TaKaRa Taq™ Version 2.0 plus dye, 2 μL of DNA template, and ddH_2_O added to a final volume of 25 μL. The thermal cycling parameters for the polymerase chain reaction included an initial denaturation step at 95 °C lasting 3 min, followed by 30 sequential cycles. Each cycle was composed of three stages: denaturation at 95 °C for 30 s, an annealing phase at 48 °C for 30 s, and an extension period at 72 °C for 1 min. After completing these cycles, a final extension step was performed at 72 °C for 7 min to ensure full product synthesis. Following the completion of the amplification process, the resulting PCR products were subjected to analysis via electrophoresis using a 2% agarose gel.

### 2.6. Whole-Genome Sequencing and Bioinformatics Analysis

Whole-genome sequencing is extensively applied for species identification, typing, and comprehensive genomic characterization of *E. faecium* isolates [17,18]. Therefore, in this study, whole-genome sequencing was performed on the isolates following PCR-based identification.

#### 2.6.1. Genomic DNA Sequencing and Library Construction

Genomic DNA was quantified using a Qubit fluorometer (Thermo Fisher Scientific, Waltham, MA, USA), and its integrity was assessed via 1% agarose gel electrophoresis (agarose from Bio-Rad, Hercules, CA, USA). Following the quantification and integrity check of the genomic DNA, sequencing libraries were then prepared by adhering to the manufacturer’s instructions with a Watchmaker DNA Library Prep Kit (Cat. No. 7K0019-096, Watchmaker Genomics, Boulder, CO, USA). To initiate this process, each sample was prepared by subjecting 200 nanograms of starting DNA to enzymatic shearing, which broke the DNA into segments with an average length of 350 base pairs. Subsequently, the resulting DNA fragments were processed through end repair, A-tailing, and adapter ligation.

Following the completion of library construction, an initial assessment of the sample quantity was carried out using a Qubit 3.0 fluorometer (Thermo Fisher Scientific, Waltham, MA, USA). This was followed by a detailed evaluation of the library’s fragment size distribution through analysis (Agilent Technologies, Santa Clara, CA, USA). Once the library size and peak profile were confirmed to meet specifications, the library was accurately quantified via qPCR using the ABI QuantStudio 12K Flex system (Thermo Fisher Scientific, Waltham, MA, USA) to ensure library quality and the accuracy of subsequent cluster generation. After passing the quality checks, these valid libraries were then subjected to sequencing on the Illumina NovaSeq 6000 platform (equipped with an S4 flow cell, Illumina, San Diego, CA, USA) with the use of a NovaSeq 6000 S4 Reagent Kit (v1.5, Illumina, San Diego, CA, USA), resulting in 150 base pair paired-end reads being generated. The sequencing process was conducted by Anoroad Co., Ltd. (Beijing, China), which utilized the Illumina NovaSeq 6000 platform with S4 flow cell and the corresponding v1.5 NovaSeq 6000 S4 Reagent Kit to produce 150 base pair paired-end sequences.

#### 2.6.2. Sequencing Data Quality Control and Filtering

Our sequencing service provider, Anoroad Co., Ltd., executed the quality control (QC) and filtering of the raw sequencing data as part of the standard bioinformatics pipeline (Beijing, China). The procedure included (1) initial quality assessment of raw reads using FastQC; (2) Adapter sequences and low-quality bases (Q-score < 20) were removed, and reads shorter than 50 bp were discarded using Trimmomatic; (3) elimination of potential host-derived contamination by aligning reads to the host reference genome using BWA and removing any aligned reads. The high-quality cleaned data resulting from this standard pipeline were then delivered to us and used for all downstream de novo assembly and analysis.

#### 2.6.3. Genome Assembly

To construct the genome sequence, the high-quality subreads obtained from filtering were processed with Canu v1.5 (https://github.com/marbl/canu; accessed on 2 March 2025) for assembly. Following this, the resulting contigs were further circularized using Circlator v1.5.5 (https://sanger-pathogens.github.io/circlator; accessed on 2 March 2025).

#### 2.6.4. Prediction of Genomic Components

Coding genes were predicted using Prodigal (v2.6.3). The GenBlastA (v1.0.4) program was used to scan the whole genomes after masking the predicted functional genes. Following this, further analysis was performed on the identified possible candidates to detect non-mature and frame-shift mutations by means of GeneWise (v2.2.0). tRNA genes were identified with tRNAscan-SE v2.0, while rRNA genes were predicted using Infernal (v1.1.3). RepeatMasker was employed for the identification of repetitive sequences. Subsequent bioinformatics analyses also included the prediction of prophage regions via PhiSpy (v2.3), the detection of CRISPR arrays through CRT (v1.2), and the identification of genomic islands using IslandPath-DIMOB (v0.2). Secondary metabolite biosynthetic gene clusters were predicted using antiSMASH (v5.0.0), and promoters were predicted using PromPredict (v1).

#### 2.6.5. Functional Annotation

To assign functional roles, the deduced amino acid sequences were searched via BLAST (https://blast.ncbi.nlm.nih.gov/Blast.cgi; accessed on 2 March 2025; with an e-value cutoff of 1 × 10^−5^) against multiple databases, which include Nr, TrEMBL, Swiss-Prot, MBL, KEGG, and eggNOG. Gene Ontology (GO) annotation was performed using Blast2GO. Furthermore, the potential pathogenicity and drug resistance profiles were investigated by querying the predicted proteins against specialized databases, such as CAZy, TCDB, CARD, and PHI.

### 2.7. Phylogenetic Analysis

Each of the single-copy gene families underwent individual alignment using MUSCLE (http://www.drive5.com/muscle/; accessed on 20 April 2025). A super alignment matrix was constructed by concatenating these alignments, which served as the input for constructing a Maximum Likelihood phylogenetic tree with RAxML (http://sco.h-its.org/exelixis/web/software/raxml/index.html; accessed on 20 April 2025).

### 2.8. Observation by Transmission Electron Microscopy (TEM)

To proceed with the subsequent experiment, the bacterial strain that had been stored in the previous section was first reactivated by the method of streaking onto blood agar plates. After the revival process, a single colony was selected and transferred into 5 mL of LB broth, which was then incubated at 37 °C for a duration of 24 h. Following this, the resulting bacterial suspension was centrifuged at 12,000 revolutions per minute for a duration of 3 min, after which the solid sediment was carefully rinsed with PBS solution. This washing process was then carried out an additional two times to ensure thorough cleaning. After the final PBS wash, the bacterial pellet was fixed using 2.5% glutaraldehyde, followed by post-fixation with 1% osmium tetroxide. Subsequent processing included dehydration and embedding in epoxy resin to prepare samples for imaging. Subsequently, ultrathin sections were stained and subsequently observed under a Hitachi HT7800 transmission electron microscope at the Experimental Technology Platform of Sichuan University (West China). Imaging was performed at an accelerating voltage of 80 kV.

### 2.9. Antimicrobial Susceptibility Testing

To assess how the isolate responds to various antibacterial agents, we employed the disk diffusion technique to evaluate its resistance or sensitivity to multiple antibiotics. To assess how responsive the isolate was to various antibiotics, a series of tests were conducted, with the results presented in Table 1. The susceptibility testing of the isolate against multiple antibiotics was conducted in accordance with the criteria set by the Clinical and Laboratory Standards Institute (CLSI) [19].

### 2.10. Mouse Challenge Experiment and Histopathological Observation

The frozen bacterial stock was revived and cultured for 24 h, after which the bacterial concentration was determined using the plate counting method. A 0.2 mL aliquot of a bacterial suspension at a concentration of 1 × 10^7^ CFU/mL was administered via oral gavage to the Kunming mice that had been housed under isolation for 10 days. The control group received 0.2 mL of sterile physiological saline via gavage (*n* = 5 per group). Following administration, the condition and mortality of the mice were observed and recorded every 12 h. Deceased mice underwent aseptic dissection, and colon tissue was collected and fixed in 4% paraformaldehyde for 48–72 h. The fixed tissue was then processed for hematoxylin and eosin (HE) staining. Stained sections were observed under a light microscope (HE staining was performed by Wuhan Boerfu Biotechnology Co., Ltd. (Wuhan, China). Additionally, colon tissue from aseptically dissected mice was used for bacterial re-isolation and identification.

### 2.11. Transcriptome Sequencing and Differential Expression Gene Analysis

At 72 h post-infection, colon tissues were collected from both the infected (with *E. faecium*) and uninfected control mice, with three mice per group. Following the susceptibility test, the collected tissues were promptly washed with pre-cooled sterile phosphate-buffered saline (pH 7.2) and then rapidly frozen in liquid nitrogen to maintain the integrity of RNA molecules. RNA extraction and subsequent RNA-seq analysis of these samples were conducted by Annoroad Gene Technology Co., Ltd. (Beijing, China).

### 2.12. Data Analysis

#### 2.12.1. Differential Expression Analysis

When biological replicates were accessible for the specimens, the differential expression analysis between the two comparison groups was conducted using DESeq2 software (version 1.44.0). This statistical tool, which is designed to analyze digital gene expression data, relies on a mathematical model based on the negative binomial distribution to identify genes with significant expression changes. To control the false discovery rate, we applied the Benjamini-Hochberg method to adjust the resulting *p*-values. Genes with an adjusted *p*-value ≤ 0.05, as identified by DESeq2 (version 1.44.0), were designated as differentially expressed.

#### 2.12.2. Differential Gene Enrichment Analysis

Differentially expressed genes underwent GO enrichment analysis and KEGG pathway enrichment statistics, with the analysis being performed via the clusterProfiler (version 4.0.0) computational tool. Differentially expressed genes underwent GO enrichment analysis and KEGG pathway enrichment statistics, with the analysis being performed via the clusterProfiler computational tool. GO terms and KEGG pathways with a *p*-value < 0.05 were considered significantly enriched.

## 3. Results

### 3.1. Isolation and Identification of Yak-Derived E. faecium

**Figure 1:**
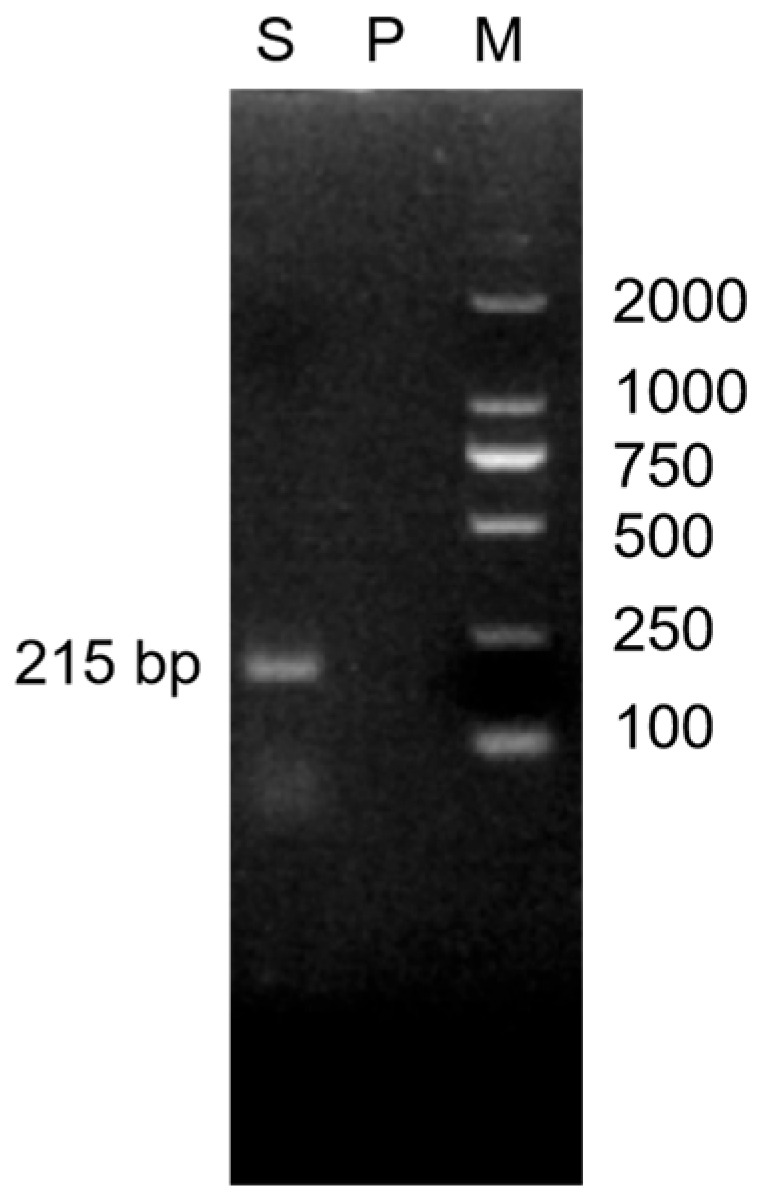
PCR amplification of *E. faecium* using species-specific primers (note: M: DNA marker; S: sample; P: negative control), (the original pictures can be found in Figure S1).

Gram-positive cocci appearing blue-violet were isolated from the yak fecal sample, consistent with the typical morphological characteristics of the genus *Enterococcus*. The isolate was biochemically characterized as catalase-negative, exhibiting growth in 6.5% NaCl and on bile esculin agar. Subsequent to this initial biochemical profiling, species confirmation was achieved through the use of PCR with primers that are specific to *E. faecium*. When the product of the PCR reaction was analyzed through electrophoresis on a 2% agarose gel, a clear target band was observed at the anticipated molecular weight (as shown in Figure 1). This result provided additional confirmation that the isolated strain belongs to the species *E. faecium*.

### 3.2. Whole-Genome Sequencing Results

**Figure 2:**
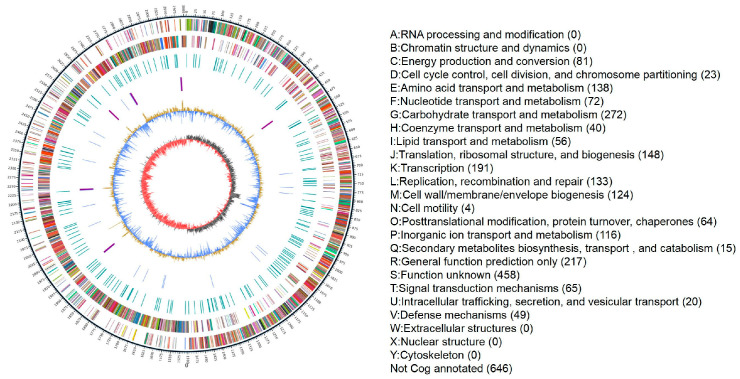
Circular genome map. (From outermost to innermost: Ring 1, genome scale (ticks at 5-kb intervals); Rings 2 and 3, coding sequences on the forward and reverse strands, respectively, colored by COG functional categories; Ring 4, repetitive sequences; Ring 5, tRNA (blue) and rRNA (purple). Ring 6, the GC content variation (light-yellow peaks above average, blue regions below average); Ring 7, GC-skew (dark gray for G > C, red for C > G).

The genome size of the isolated *E. faecium* strain was 3,008,203 bp, with a GC content of 38.05%. The average sequencing depth was 1494×, and the N50 was 16,574 bp. Four prophage sequences were identified, while no plasmid sequences were detected (a Circos plot visualizing the genome is presented in Figure 2).

A total of three categories of antimicrobial resistance genes were predicted, including the aminoglycoside resistance gene *AAC(6′)-Ii*, the macrolide and streptogramin B resistance gene *msrC*, and the lincosamide–streptogramin A–pleuromutilin resistance gene *eatAv*.

Furthermore, three categories of virulence system genes were detected, such as the collagen-binding adhesin gene *Acm*, the transcriptional regulator gene *bopD*, and the ATP-dependent protease system gene *ClpP* (E-value < 1 × 10^−10^, percent identity ≥80%, Query Cover ≥90%).

### 3.3. Phylogenetic Tree Analysis

**Figure 3:**
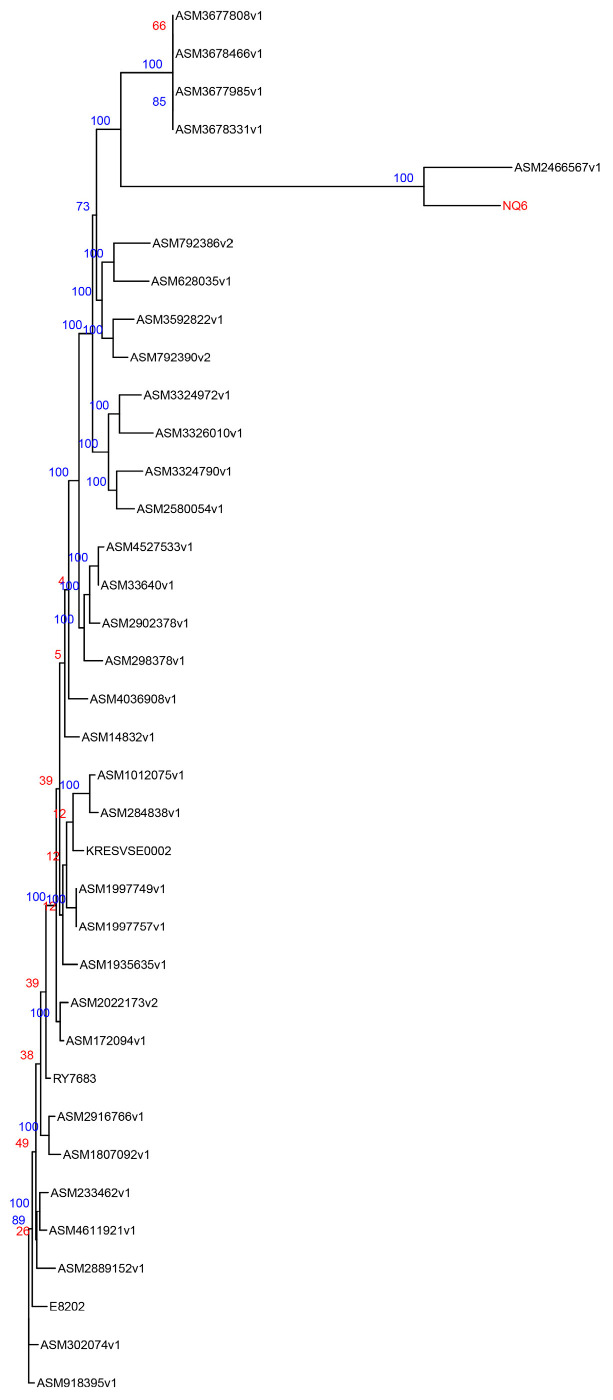
Phylogenetic tree based on genome assembly results (The numbers represent support values, and the labels in red font (NQ6) indicate the experimental strains).

Phylogenetic analysis revealed that the yak-derived strain NQ6 shares the closest genetic relationship with the human-derived strain ASM246656v1, suggesting that these two strains may share a common lineage (Figure 3).

### 3.4. Transmission Electron Microscopy (TEM) Observation Results

**Figure 4:**
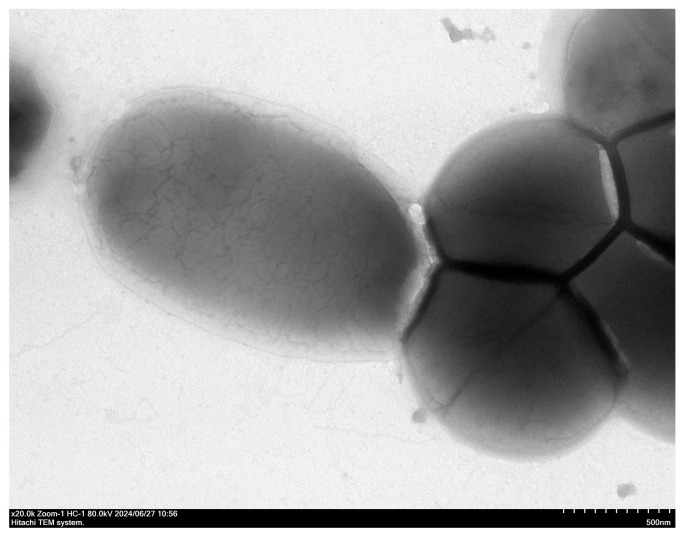
Representative TEM showing *E. faecium* (×20,000).

TEM images revealed the isolate to be spherical or ovoid cocci arranged in short chains. The cells exhibited a smooth outer layer without any flagella observed; conversely, a possible capsule was visible (Figure 4).

### 3.5. Antimicrobial Susceptibility Testing Results

The isolated *E. faecium* strain demonstrated resistance to β-lactams (penicillin, to which *E. faecium* is intrinsically resistant), aminoglycosides (kanamycin), macrolides (erythromycin), and lincosamides (clindamycin) (Table 2).

### 3.6. In Vivo Challenge and Pathological Evaluation in Mice

**Figure 5:**
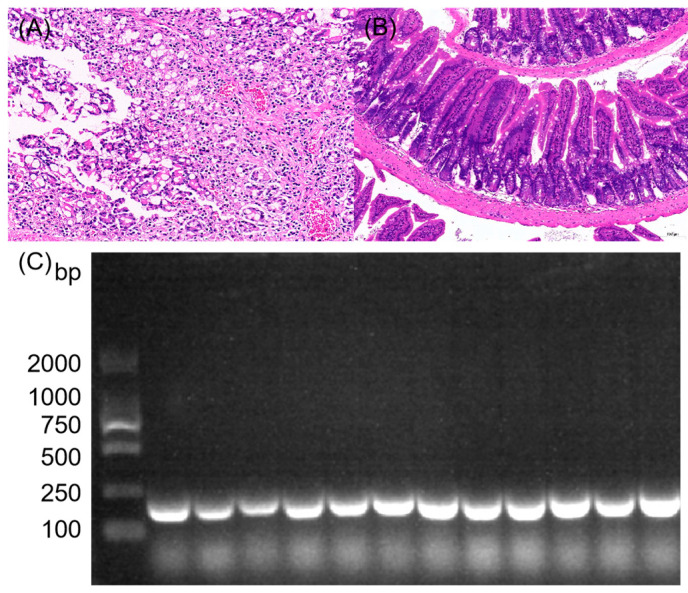
Characterization of infection in a mouse model. (**A**) HE image showing pathological changes in the colon of a mouse that succumbed to infection (200×). (**B**) HE image showing normal colon histology in a control mouse (100×). (**C**) PCR electrophoresis results demonstrating the specific presence of the inoculated strain in colonic tissues from infected mice, (the original pictures can be found in Figure S2).

The infected group displayed a range of clinical symptoms, beginning as early as 1 day and continuing up to 10 days post-challenge, including reduced food intake, ruffled fur, and decreased activity. No abnormalities were observed in the control group. Deaths began to occur 48 h post-inoculation, with a total of two mortalities recorded within 7 days. HE staining results (Figure 5) revealed moderate structural abnormalities in the colon tissue of the infected group. This was characterized by extensive necrosis and exfoliation of mucosal epithelial cells, marked vascular congestion in the mucosal layer, and significant inflammatory cell infiltration. The control group, in contrast, maintained a normal colonic architecture. For bacterial isolation, colon tissue collected during aseptic necropsy was directly inoculated onto Columbia blood agar and incubated at 37 °C for 16 h. Bacterial re-isolates were identified using the PCR method described in Section 2.5. Agarose gel electrophoresis (2%) confirmed that the isolated bacteria were *E. faecium*, as indicated by bands of the expected size.

### 3.7. Transcriptome Sequencing Results

In the *E. faecium*-infected group, the proportion of high-quality sequences after filtering relative to the original raw sequences was 97.61%. After filtering, for the sequences processed in this manner, the percentage of bases achieving a quality score exceeding 30 (which corresponds to an error rate below 0.1%) in all sequences was 95.04% of the total bases. In the control group, the proportion of high-quality sequences after filtering relative to the original raw sequences was 96.56%, and the percentage of bases scoring above 30 (with an error rate below 0.1%) after filtering accounted for 95.70% of all total bases.

Following this, the filtered sequences were aligned against the reference genome, revealing a high level of alignment effectiveness. The average mapping rate in the control group was 96.49% (specifically, Control1: 97.23%; Control2: 97.04%; and Control3: 95.21%). The average mapping rate in the challenged/infected (NQ6) group was 94.68% (specifically, NQ6 (1): 95.72%; NQ6 (2): 96.52%; and NQ6 (3): 91.80%).

#### 3.7.1. Statistics of Differentially Expressed Genes

**Figure 6:**
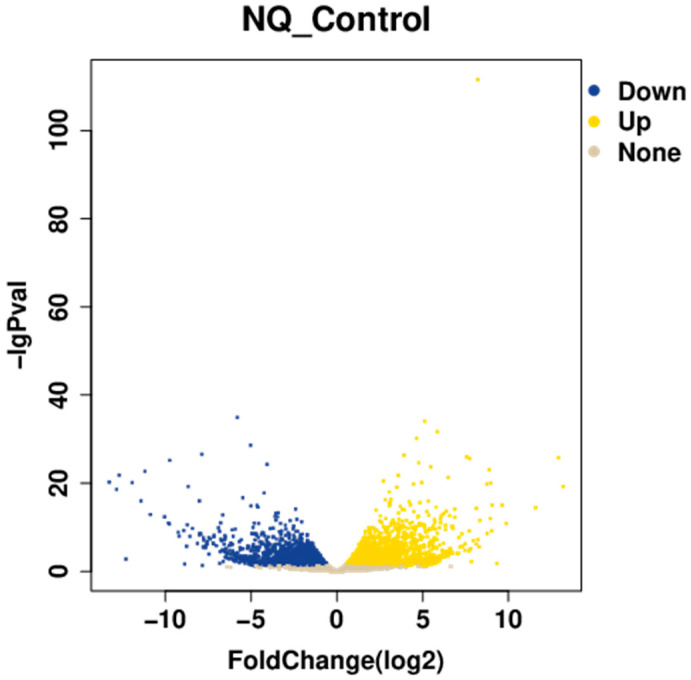
Volcano plot of differentially expressed genes.

In the colon tissues, researchers found a total of 5225 significantly different genes that were expressed, with 2621 showing increased activity and 2604 exhibiting decreased expression levels. The distribution of these differentially expressed genes is visually represented in the volcano plot depicted in Figure 6.

#### 3.7.2. GO Enrichment Analysis

**Figure 7:**
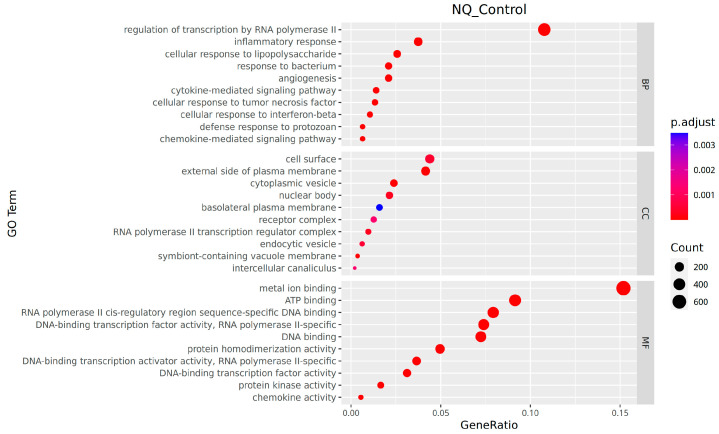
GO enrichment bubble plot.

In the molecular function classification, the genes with notable differential expression were predominantly involved in functions like sequence-specific DNA binding to RNA polymerase II cis-regulatory regions, DNA-binding transcription activator activity specific to RNA polymerase II, DNA-binding transcription factor activity, and RNA polymerase II-specific activity. In the context of cellular components, the genes with significant differential expression predominantly belong to enriched categories, including the exterior surface of the plasma membrane, symbiont-containing vacuole membrane, and cytoplasmic vesicle. Regarding biological processes, significantly differentially expressed genes were notably enriched in the regulation of transcription by RNA polymerase II, inflammatory response, and cellular response to interferon-beta (Figure 7).

As shown in Table 3, the upregulated significantly differentially expressed genes were mainly abundant in pathways or categories like the “aminoacyl-tRNA synthetase family” (e.g., the *Cars* gene), “metabolism-associated enzymes” (e.g., *Acacb*), and the “ABC transporter family” (e.g., *Abcb1b*). Conversely, the downregulated significantly differentially expressed genes showed enrichment in the “ABC transporter family” (e.g., *Abcc2*), “metabolism-associated enzymes” (e.g., *Ahcy*), and “predicted genes/pseudogenes” (e.g., *Gm5540*).

#### 3.7.3. KEGG Pathway Analysis

**Figure 8:**
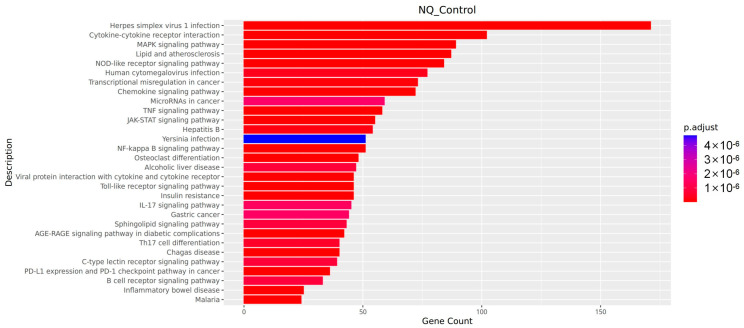
KEGG enrichment bar chart.

As shown in Figure 8, the KEGG pathway enrichment analysis indicated that the genes with altered expression levels were markedly involved in pathways closely linked to intestinal infection and immune responses. These pathways can be functionally categorized into three main groups: (1) Immune Recognition and Early Response Pathways: This category includes the Toll-like receptor signaling pathway and the NOD-like receptor signaling pathway. (2) Inflammatory Signal Transduction Pathways: These biological processes involve multiple signaling cascades, such as the activation of NF-kappa B, the MAPK pathway, the TNF signaling pathway, the interaction between cytokines and their receptors, and the signaling mechanism of chemokines. (3) Gut Infection-Specific Pathway: This refers to the inflammatory bowel disease pathway. All the pathways listed above met the criterion for significant enrichment (*p* < 0.05).

## 4. Discussion

### 4.1. Resistance and Virulence Risks Revealed by Genomic Characterization

Whole-genome sequencing analysis of the *E. faecium* isolate in this study revealed the potential genetic basis of its multidrug resistance, identifying three key resistance genes: *AAC(6′)-Ii*, *msrC*, and *eatAv*. These genes confer resistance through distinct mechanisms: *AAC(6′)-Ii* prevents antibiotic binding to the bacterial ribosomal target site via acetylation [20]; *msrC* reduces intracellular drug concentration through an efflux pump mechanism [21]; and *eatAv* mediates cross-resistance to multiple antibiotics by methylating 23S rRNA [22]. The coexistence of these three genes within a single genome forms a functionally complementary resistance network. This synergistic effect may significantly enhance the strain’s adaptability and survival advantage in environments with combination antibiotic therapy. This finding provides strong genetic evidence explaining the strain’s potential multidrug-resistant phenotype and holds important significance for drug selection in clinical anti-infective therapy. Although no high-risk *vanA/vanB* genes were detected, it is important to recognize that the genus *Enterococcus* (e.g., species carrying *vanC*) remains a reservoir for low-level glycopeptide tolerance [23].

Whole-genome sequencing predicted three key virulence genes: *bopD*, *Acm*, and *ClpP*. Among them, *bopD* promotes deep tissue invasion of the pathogen by regulating the expression of downstream virulence factors [24]. *Acm*, as a precursor of a collagen-binding adhesin, can interact strongly with type I collagen and shows weaker affinity for type IV collagen [25,26]. The *ClpP* protease system specifically targets and degrades misfolded proteins and short-lived regulatory proteins to prevent their accumulation and subsequent cytotoxicity [27]. The combination of these genes, which are linked to tissue invasion, adhesion, and stress adaptation, suggests a genetic framework that may support a pathogenic process, ranging from environmental sensing to persistent infection. However, this proposed model needs to be validated through future phenotypic and functional studies focused on this specific strain.

### 4.2. Validation of Consistency Between Phenotype and Genotype

Transmission electron microscopy observation revealed that this strain possesses a distinct capsule structure but no flagella. This non-flagellated phenotype suggests that the strain may lack the capacity for active motility and invasion. Consequently, its pathogenic strategy is more likely to rely on firm adhesion and colonization on host surfaces. This aligns with the surface adhesin genes identified, providing a molecular basis for the observed phenotype. Furthermore, may synergize with adhesion: initial attachment mediated by *Acm* offers a foothold, while the capsule protects against phagocytosis, facilitating sustained colonization and biofilm formation.

Antimicrobial susceptibility testing confirmed that the strain is resistant to penicillin (penicillin is an antibiotic with intrinsic resistance in *E. faecium* [28]), kanamycin, erythromycin, and clindamycin. Predictive analysis of the whole-genome sequence revealed the presence of resistance genes strongly aligned with the observed phenotypic resistance. Specifically, the prediction of the aminoglycoside resistance gene *AAC(6′)-Ii* provides a plausible genetic explanation for the kanamycin resistance phenotype. Similarly, the coexistence of the efflux pump gene *msrC* and the ribosomal methyltransferase gene *eatAv* predicts, at the genetic level, its cross-resistance mechanism to erythromycin and clindamycin. These correlations between genotype and phenotype indicate that the predicted resistance genes are likely functionally active. Notably, the resistance gene profile of this strain, such as *AAC(6′)-Ii* and *msrC*, is also frequently reported in E. faecium of animal origin in Europe [29], further indicating the potential transmission risk of its resistance traits.

### 4.3. Combined Analysis of HE Pathological Phenotype and Transcriptome Enrichment Results

The histopathology revealed intestinal inflammation, mucosal necrosis, vascular congestion, and immune infiltration in challenged mice.

Transcriptomic analysis explained these changes: enrichment in Toll-like and NOD-like receptor pathways indicates pathogen recognition [28,30,31,32], Subsequently, followed by activation of NF-κB and MAPK signaling promoting cytokine productions [33,34]. Chemokine pathway activation likely mediated immune cell recruitment [35,36], corroborating the observed congestion and infiltration. Additionally, immune-cell-derived enzymes may contribute to mucosal damage. Notably, the enrichment of pathways related to inflammatory bowel disease links the molecular mechanisms of the enteritis induced in this study to those of human chronic intestinal diseases, enhancing the clinical relevance of the research.

The GO analysis results corroborate the KEGG pathway findings: the significant upregulation of genes associated with aminoacyl-tRNA synthetase and ABC transporters indicates that both host cells and immune cells are in a highly activated state, requiring substantial protein synthesis and remodeling of substance transport to combat the infection [37,38]. This functionally supports the predictions derived from the aforementioned pathways.

In summary, this study predicts a “Recognition–Response–Damage” axis in *E. faecium*-induced enteritis, providing a mechanistic perspective and a basis for future targeted interventions.

## 5. Conclusions

This study successfully isolated and identified a strain of *E. faecium* from the feces of plateau yaks; the bacterium’s genomic characteristics suggest its potential public health relevance. Phylogenetic analysis revealed a close genetic relationship between this yak-derived strain and human clinical isolates. This molecular evidence supports a theoretical risk of *E. faecium* cross-host transmission between livestock environments and human populations. Furthermore, through the correlation analysis of genotype and phenotype, this study confirmed that the strain not only carries resistance genes, including *AAC(6′)-Ii*, *msrC*, and *eatAv*, but also exhibits an actual resistant phenotype to antibiotics, such as penicillin, kanamycin, erythromycin, and clindamycin. Simultaneously, its genome contains key virulence genes, such as *bopD*, *Acm*, and *ClpP*. In vivo experiments further validated its pathogenic potential, and by integrating transcriptomic analysis, we fully elucidated the “Recognition–Response–Damage” molecular pathway through which it mediates intestinal inflammation, mechanistically explaining its pathogenicity. In summary, this work not only reports a yak-derived *E. faecium* strain with multidrug resistance and pathogenic potential but, more importantly, also systematically assesses its potential public health risk from three dimensions: “cross-host transmission risk, reservoir of resistance and virulence genes, and in vivo pathogenic mechanisms.” This finding lays the groundwork for developing targeted surveillance and control strategies, highlighting the importance of judicious antimicrobial use to mitigate the potential enrichment and spread of such strains.

## Figures and Tables

**Table 1:** Antibiotics used in the susceptibility test.

Antibiotic	Dosage
Penicillin	10 U
Gentamicin	120 μg
Kanamycin	30 μg
Cefazolin	30 μg
Ceftazidime	30 μg
Cephradine	30 μg
Vancomycin	30 μg
Erythromycin	15 μg
Tetracycline	30 μg
Ofloxacin	30 μg
Clindamycin	30 μg
Furazolidone	30 μg

**Table 2:** Antimicrobial susceptibility testing results by the disk diffusion method.

Antimicrobial Agent	Content (μg/Disk)	Breakpoints (mm)			Inhibition Zone Diameter (mm)	Susceptibility
		R	I	S		
Penicillin	10 U	≤14	14–15	≥15	0	R
Gentamicin	120	≤6	7–9	≥10	9	I
Kanamycin	30	≤13	14–17	≥18	7	R
Cefazolin	30	≤14	15–17	≥18	24	S
Ceftazidime	30	≤14	15–17	≥18	22	S
Cephradine	30	≤14	15–17	≥18	18	S
Vancomycin	30	≤14	15–16	≥17	17	S
Erythromycin	15	≤13	14–22	≥23	12	R
Tetracycline	30	≤14	15–18	≥19	18	I
Ofloxacin	5	≤12	13–15	≥16	23	S
Clindamycin	2	≤14	15–20	≥21	0	R
Furazolidone	300	≤14	15–16	≥17	15	

Abbreviations: R, resistant; I, intermediate; S, susceptible.

**Table 3:** Upregulated/downregulated significantly differentially expressed genes.

Gene Category	Gene Names	Functional Category
Upregulated	*Cars*, *Gars*, *Lars*, *Tars*, *Mars1*, *Aars*, *Nars*, *Etf1*	Aminoacyl-tRNA synthetase family
	*Abcb1b*, *Abcc4*, *Abcc1*	ABC transporter family
	*Chd7*, *Chd2*	Transcription and epigenetic regulation
	*Acacb*, *Shmt2*, *Gbe1*	Metabolism-related enzymes
	*Myo5a*	Cytoskeleton and motility
	*Ppp3cc*	Signal transduction
	*Rpl3-ps1*	Predicted gene/pseudogene
Downregulated	*Gm5540*, *Gm6682*, *Gm3756*, *Gm5837*, *Gm9826*	Predicted genes/pseudogenes
	*Abcc2*	ABC transporter family
	*Ahcy*, *Aacs*	Metabolism-related enzymes
	*Acta1*	Cytoskeleton and motility

## Data Availability

The data used in this research are publicly accessible through the NCBI SRA database at the URL https://www.ncbi.nlm.nih.gov/sra/, which was accessed on 18 July 2025. The relevant reference identifiers for the data are SUB15406315, SAMN49535451, and additional numbers 2 through 6.

## Supplementary Materials

The following supporting information can be downloaded at https://www.mdpi.com/article/10.3390/vetsci13010103/s1: Figures S1 and S2: Original images of PCR electrophoresis results.

## References

[1] Corredor N.C., López C., Aguilera P.A., Prieto L.M., Rodríguez-Leguizamón G., Leal A.L., Ovalle-Guerro M.V., Pardo-Oviedo J.M., Chica C.E., Patarroyo M.A. (2019). An epidemiological and molecular study regarding the spread of vancomycin-resistant Enterococcus faecium in a teaching hospital in Bogotá, Colombia 2016. BMC Infect. Dis..

[2] Udaondo Z., Abram K., Kothari A., Jun S.R. (2023). Top-Down Genomic Surveillance Approach To Investigate the Genomic Epidemiology and Antibiotic Resistance Patterns of *Enterococcus faecium* Detected in Cancer Patients in Arkansas. Microbiol. Spectr..

[3] Kwentoh I., Henry T. (2023). Look Beyond Syncope: A Positive Outcome in the Management of Multiple Bladder Diverticuli-Associated *Enterococcus faecium* Urinary Tract Infection. Cureus.

[4] Giannakopoulos X., Sakkas H., Ragos V., Tsiambas E., Bozidis P., Evangelou A.M., Papadopoulou C., Petrogiannopoulos L., Sofikitis N. (2019). Impact of enterococcal urinary tract infections in immunocompromised-neoplastic patients. J. BUON.

[5] Brinkwirth S., Feig M., Noll I., Eckmanns T., Dörre A., Haller S., Willrich N. (2025). Changing dynamics of bloodstream infections due to methicillin-resistant *Staphylococcus aureus* and vancomycin-resistant *Enterococcus faecium* in Germany, 2017–2023: A continued burden of disease approach. Antimicrob. Resist. Infect. Control..

[6] Alotaibi G., Khan K., Al Mouslem A.K., Ahmad Khan S., Naseer Abbas M., Abbas M., Ali Shah S., Jalal K. (2022). Pan genome based reverse vaccinology approach to explore *Enterococcus faecium* (VRE) strains for identification of novel multi-epitopes vaccine candidate. Immunobiology.

[7] Chen V., Griffin M.E., Maguin P., Varble A., Hang H.C. (2021). RecT Recombinase Expression Enables Efficient Gene Editing in *Enterococcus* spp. Appl. Environ. Microbiol..

[8] Freitas A.R., Pereira A.P., Novais C., Peixe L. (2021). Multidrug-resistant high-risk *Enterococcus faecium* clones: Can we really define them?. Int. J. Antimicrob. Agents.

[9] Tumpa A., Štritof Z., Pintarić S. (2022). Prevalence and antimicrobial susceptibility of *Enterococcus* spp. from urine of dogs and cats in northwestern Croatia. Res. Vet. Sci..

[10] Thu W.P., Sinwat N., Bitrus A.A., Angkittitrakul S., Prathan R., Chuanchuen R. (2019). Prevalence, antimicrobial resistance, virulence gene, and class 1 integrons of *Enterococcus faecium* and *Enterococcus faecalis* from pigs, pork and humans in Thai-Laos border provinces. J. Glob. Antimicrob. Resist..

[11] Samad M.A., Sagor M.S., Hossain M.S., Karim M.R., Mahmud M.A., Sarker M.S., Shownaw F.A., Mia Z., Card R.M., Agunos A. (2022). High prevalence of vancomycin non-susceptible and multi-drug resistant enterococci in farmed animals and fresh retail meats in Bangladesh. Vet. Res. Commun..

[12] Beukers A.G., Zaheer R., Goji N., Amoako K.K., Chaves A.V., Ward M.P., McAllister T.A. (2017). Comparative genomics of Enterococcus spp. isolated from bovine feces. BMC Microbiol..

[13] Kwit R., Zając M., Śmiałowska-Węglińska A., Skarżyńska M., Bomba A., Lalak A., Skrzypiec E., Wojdat D., Koza W., Mikos-Wojewoda E. (2023). Prevalence of Enterococcus spp. and the Whole-Genome Characteristics of *Enterococcus faecium* and *Enterococcus faecalis* Strains Isolated from Free-Living Birds in Poland. Pathogens.

[14] Soltani S., Fallah T., Shafiei M., Shahraki A.H., Iranbakhsh A. (2025). Investigating the prevalence of CRISPR-Cas system and their association with antibiotic resistance genes and virulence factors in *Enterococcus faecalis* and *Enterococcus faecium* strains isolated from hospitalized patients. J. Glob. Antimicrob. Resist..

[15] Alduhaidhawi A.H.M., AlHuchaimi S.N., Al-Mayah T.A., Al-Ouqaili M.T.S., Alkafaas S.S., Muthupandian S., Saki M. (2022). Prevalence of CRISPR-Cas Systems and Their Possible Association with Antibiotic Resistance in *Enterococcus faecalis* and *Enterococcus faecium* Collected from Hospital Wastewater. Infect. Drug Resist..

[16] Iweriebor B.C., Obi L.C., Okoh A.I. (2015). Virulence and antimicrobial resistance factors of *Enterococcus* spp. isolated from fecal samples from piggery farms in Eastern Cape, South Africa. BMC Microbiol..

[17] Stellfox M.E., Van Tyne D. (2022). Last Bacteria Standing: VREfm Persistence in the Hospitalized Gut. mBio.

[18] Van Hal S.J., Willems R.J.L., Gouliouris T., Ballard S.A., Coque T.M., Hammerum A.M., Hegstad K., Westh H.T., Howden B.P., Malhotra-Kumar S. (2021). The global dissemination of hospital clones of Enterococcus faecium. Genome Med..

[19] Schuetz A.N., Ferrell A., Hindler J.A., Humphries R., Bobenchik A.M. (2025). Overview of changes in the Clinical and Laboratory Standards Institute Performance Standards for Antimicrobial Susceptibility Testing: M100 32nd and 33rd editions. J. Clin. Microbiol..

[20] Prabhu D., Shankari G., Rajamanikandan S., Jeyakanthan J., Velusamy P., Gopinath S.C.B., Pattabi S. (2024). Designing potential lead compounds targeting aminoglycoside N (6′)-acetyltransferase in *Serratia marcescens*: A drug discovery strategy. Int. J. Biol. Macromol..

[21] Chen W., Wang Q., Wu H., Xia P., Tian R., Li R., Xia L. (2024). Molecular epidemiology, phenotypic and genomic characterization of antibiotic-resistant enterococcal isolates from diverse farm animals in Xinjiang, China. Sci. Total Environ..

[22] Fatoba D.O., Amoako D.G., Akebe A.L.K., Ismail A., Essack S.Y. (2022). Genomic analysis of antibiotic-resistant *Enterococcus* spp. reveals novel enterococci strains and the spread of plasmid-borne Tet(M), Tet(L) and Erm(B) genes from chicken litter to agricultural soil in South Africa. J. Environ. Manag..

[23] Zakaria N.D., Hamzah H.H., Salih I.L., Balakrishnan V., Abdul Razak K. (2023). A Review of Detection Methods for Vancomycin-Resistant *Enterococci* (VRE) Genes: From Conventional Approaches to Potentially Electrochemical DNA Biosensors. Biosensors.

[24] Zhang L., Wang F., Jia L., Yan H., Gao L., Tian Y., Su X., Zhang X., Lv C., Ma Z. (2023). *Edwardsiella piscicida* infection reshapes the intestinal microbiome and metabolome of big-belly seahorses: Mechanistic insights of synergistic actions of virulence factors. Front Immunol..

[25] Nallapareddy S.R., Weinstock G.M., Murray B.E. (2003). Clinical isolates of *Enterococcus faecium* exhibit strain-specific collagen binding mediated by Acm, a new member of the MSCRAMM family. Mol. Microbiol..

[26] Nallapareddy S.R., Singh K.V., Murray B.E. (2006). Construction of improved temperature-sensitive and mobilizable vectors and their use for constructing mutations in the adhesin-encoding acm gene of poorly transformable clinical *Enterococcus faecium* strains. Appl. Environ. Microbiol..

[27] Ghanbarpour A., Zhang J.J., Davis J.H., Baker T.A., Sauer R.T. (2025). Structural insights into the *Pseudomonas aeruginosa* ClpP1•ClpP2 heterocomplex and its interactions with the AAA+ ClpX unfoldase. Protein Sci..

[28] Hunashal Y., Kumar G.S., Choy M.S., D’Andréa É.D., Da Silva Santiago A., Schoenle M.V., Desbonnet C., Arthur M., Rice L.B., Page R. (2023). Molecular basis of β-lactam antibiotic resistance of ESKAPE bacterium *E. faecium* Penicillin Binding Protein PBP5. Nat. Commun..

[29] Dec M., Stępień-Pyśniak D., Gnat S., Fratini F., Urban-Chmiel R., Cerri D., Winiarczyk S., Turchi B. (2020). Antibiotic Susceptibility and Virulence Genes in Enterococcus Isolates from Wild Mammals Living in Tuscany, Italy. Microb. Drug Resist..

[30] Jang K.K., Heaney T., London M., Ding Y., Putzel G., Yeung F., Ercelen D., Chen Y.H., Axelrad J., Gurunathan S. (2023). Antimicrobial overproduction sustains intestinal inflammation by inhibiting Enterococcus colonization. Cell Host Microbe.

[31] Kaur A., Kaushik D., Piplani S., Mehta S.K., Petrovsky N., Salunke D.B. (2021). TLR2 Agonistic Small Molecules: Detailed Structure-Activity Relationship, Applications, and Future Prospects. J. Med. Chem..

[32] Kawai T., Ikegawa M., Ori D., Akira S. (2024). Decoding Toll-like receptors: Recent insights and perspectives in innate immunity. Immunity.

[33] Tian H., Ling N., Guo C., Gao M., Wang Z., Liu B., Sun Y., Chen Y., Ji C., Li W. (2024). Immunostimulatory activity of sea buckthorn polysaccharides via TLR2/4-mediated MAPK and NF-κB signaling pathways in vitro and in vivo. Int. J. Biol. Macromol..

[34] Lin X., Guo X., Qu L., Tu J., Li S., Cao G., Liu Y. (2022). Preventive effect of Atractylodis Rhizoma extract on DSS-induced acute ulcerative colitis through the regulation of the MAPK/NF-κB signals in vivo and in vitro. J. Ethnopharmacol..

[35] Dong D., Su T., Chen W., Wang D., Xue Y., Lu Q., Jiang C., Ni Q., Mao E., Peng Y. (2023). *Clostridioides difficile* aggravates dextran sulfate solution (DSS)-induced colitis by shaping the gut microbiota and promoting neutrophil recruitment. Gut Microbes.

[36] Zheng C., Li J., Chen H., Ma X., Si T., Zhu W. (2024). Dual role of CD177 + neutrophils in inflammatory bowel disease: A review. J. Transl. Med..

[37] Shim J.A., Jo Y., Hwang H., Lee S.E., Ha D., Lee J.H., Kim J., Song P., Lee D., Hong C. (2022). Defects in aminoacyl-tRNA synthetase cause partial B and T cell immunodeficiency. Cell Mol. Life Sci..

[38] De Waal T., Handin N., Brouwers J., Ferrante M., Vermeire S., Vanuytsel T., Artursson P., Augustijns P. (2022). The impact of inflammation on the expression of drug transporters and metabolic enzymes in colonic tissue from ulcerative colitis patients. Int. J. Pharm..

