# Supplementary figures and images for: Characterization of Enterococcus faecium Based on Multi-Omics Approaches: Genomic, Transcriptomic, and Phenotypic Analyses
Source: Vet Sci. 2026 Jan 21;13(1):103. doi: 10.3390/vetsci13010103 (PMC12846527; doi:10.3390/vetsci13010103)

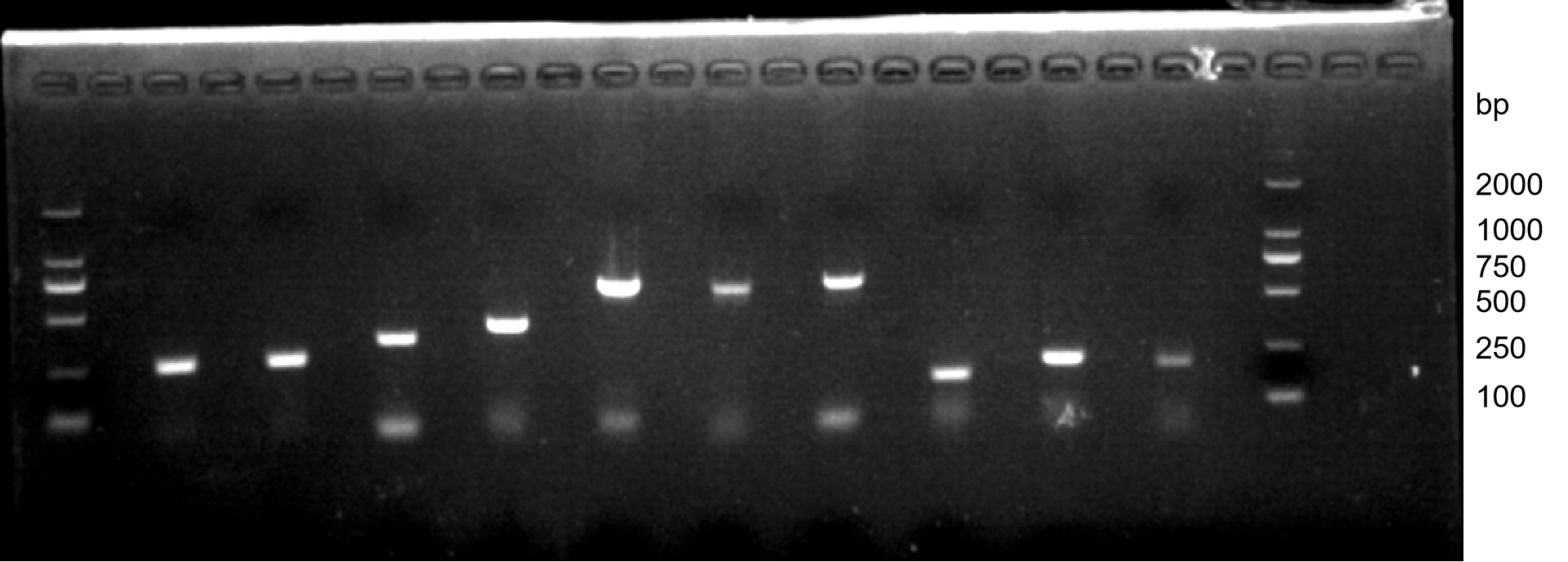

Supplement: Supplementary file 1 [file vetsci-13-00103-s001.zip › vetsci-4064343,original_images/Figure S1 .png]

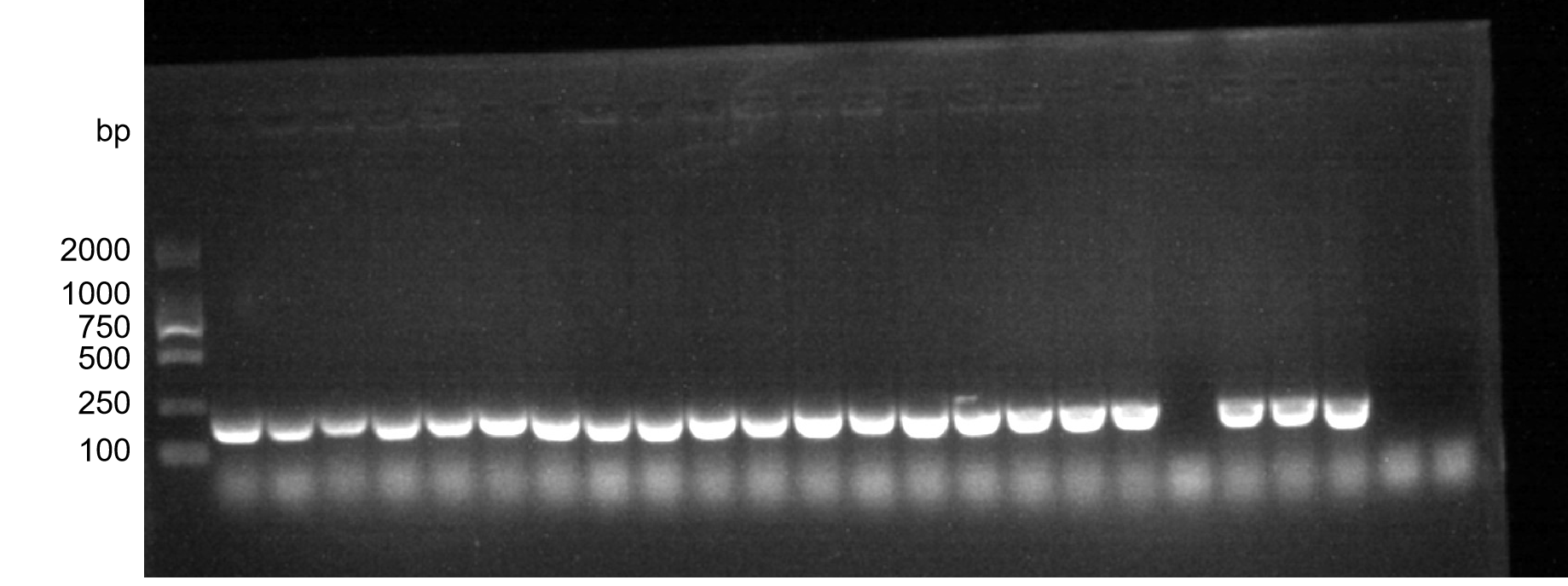

Supplement: Supplementary file 1 [file vetsci-13-00103-s001.zip › vetsci-4064343,original_images/Figure S2 .png]
